# The Meaning of Screening: Exploring user experience of an aneuploidy screening educational game

**DOI:** 10.1002/jgc4.70183

**Published:** 2026-02-18

**Authors:** Naomi O. Riches, Erin P. Johnson, Akila Subramaniam, Neeta L. Vora, Kimberly A. Kaphingst, Jenna Junge, Malique Valle, Erin Rothwell

**Affiliations:** ^1^ Department of Obstetrics and Gynecology, Spencer Fox Eccles School of Medicine University of Utah Salt Lake City Utah USA; ^2^ Department of Obstetrics and Gynecology, School of Medicine University of Alabama at Burmingham Birmingham Alabama USA; ^3^ Department of Genetics University of North Carolina Chapel Hill North Carolina USA; ^4^ Department of Communication and Huntsman Cancer Institute University of Utah Salt Lake City Utah USA; ^5^ Department of Chemistry University of Utah Salt Lake City Utah USA

**Keywords:** aneuploidy screening, cfDNA, health education games, informed decision‐making, NIPT, prenatal education

## Abstract

The Meaning of Screening is an interactive, gamified educational web‐based app designed to support aneuploidy screening decision‐making and was created through interviews and iterative development with patients, providers, and experts. The goal of this qualitative study is to summarize users' game experience, including how the app impacted their decision‐making process. For this study, participants recruited into the randomized controlled trial to assess the effectiveness of the app on informational needs, preferences, and shared decision‐making of patients' aneuploidy screening decision were interviewed about their experience with the game. Participants were selected through a two‐step process: opting into interviews followed by sampling to achieve population representativeness. Inductive content analysis was conducted on the transcripts, with codes created from the transcribed interview content, where the interviewee's words were used as descriptors. Seventeen participants were interviewed about their experience with and opinions about the app titled “Meaning of Screening.” Over half of the participants were aware of aneuploidy screening prior to interacting with the game (*n* = 13). Three major categories were identified through inductive content analysis: Impact, User Experience, and Integration into Clinical settings. Participants shared their experience with the game, with many expressing it helped to increase knowledge and understanding, assist values clarification, support decision‐making factors, and promote shared decision‐making with their partner and healthcare provider. Healthcare professionals play a crucial role in patients' aneuploidy screening decision‐making by offering accurate, thorough, and unbiased information about available options. However, during prenatal visits, the provider has competing topics to discuss. When limited time doesn't allow for proficient education in the clinic, it is important that patients obtain accurate information through other means. This allows the patient to integrate this knowledge with their values when considering the potential implications of undergoing aneuploidy screening. Results from this study support the Meaning of Screening app can provide additional education to support decision‐making about aneuploidy screening.

**What is known about this topic**

During each prenatal visit, healthcare providers discuss many topics with patients, which limits the time available to educate patients about aneuploidy screening. Decision support tools can help providers increase patients' knowledge about a health topic, incorporate values clarification, and facilitate shared decision‐making on various health topics.

**What this paper adds to the topic**

The Meaning of Screening app was created to support aneuploidy screening decision‐making. Results from this study indicate that this app can provide additional education to support this medical decision‐making process.

## 1 INTRODUCTION

Aneuploidy screening, also referred to as cell‐free DNA screening (cfDNA) or noninvasive prenatal testing (NIPT), is a blood test to screen for a pregnant woman's risk for certain genetic conditions, such as Down Syndrome and Trisomy 18. The American College of Obstetrics and Gynecology (ACOG) recommends aneuploidy screening for all patients, regardless of their age or medical history (ACOG, n.d.). However, while prenatal cfDNA is the most accurate among noninvasive screening tests, there is a small risk of false‐positives, and results are not necessarily easily understood by those unfamiliar with risk estimates. Those receiving a high‐risk result should be offered confirmatory testing, either prenatally or after birth (Liehr, 2022), as well as personalized genetic counseling. If a patient does not understand, or is not able to interpret, the potential results and implications from the screening test, they may experience unnecessary stress or choose to terminate before diagnostic confirmation (ACOG, 2008; Kliff & Bhatia, 2022).

Pregnant women generally attend their first prenatal visit with limited knowledge about cfDNA (Dane et al., 2018; Farrell et al., 2020) and are not aware of the implications of screening results (Agatisa et al., 2015; Piechan et al., 2016). Farrell et al. (2021) asked participants to complete a survey about their knowledge and decision‐making preferences regarding carrier screening and cfDNA prior to their first prenatal visit, and participants' limited knowledge presented a barrier to decision‐making (Farrell et al., 2021). Participants also reported a preference to learn about a range of conditions prior to completing the screening test, which presents a challenge for providers who are expected to educate patients in an increasingly complicated genetic landscape.

Obstetrics providers often lack the time to introduce cfDNA to their patients but understand that additional education is necessary in order to make an informed decision (Claesen‐Bengtson et al., 2025; Kater‐Kuipers et al., 2018; Ngan et al., 2017). Additionally, prenatal care may be provided by several different medical professionals, including obstetricians, midwives, nurse practitioners, and family medicine doctors, complicating the standardization of workflow and education. It is important for anyone offering these tests to emphasize the difference between a screening test such as cfDNA and a diagnostic test such as amniocentesis, so patients are fully informed of the limitations of the screening test. One study conducted with providers in the Netherlands (Kater‐Kuipers et al., 2018) revealed that a common misconception provided during pre‐test counseling was that cfDNA has a high enough accuracy to be a diagnostic procedure, contributing to the confusion about the difference between screening and diagnostic tests. This misunderstanding can have larger implications in how providers counsel patients confronted with a high‐risk result, which led to the 2022 United States Food and Drug Administration safety communication that warned of the risk of false positive results and the potential for inappropriate clinical decisions based solely on the results of screening (Zhan, 2022).

Therefore, it is important that patients are provided with accurate information about the screening test and related diagnostic tests, especially when faced with unfamiliar concepts (e.g., false‐positive results and probability) and additional decision‐making concerning follow‐up diagnostic testing (Labonté et al., 2019). However, the amount of information recommended to be discussed at the first prenatal visit in the United States has increased over time (Dyer et al., 2018; Gammon et al., 2016). Over the years, ACOG has recommended over 70 topics to be discussed during that visit (Christopher et al., 2022). Additionally, the COVID‐19 pandemic reduced the frequency of prenatal visits, a change that has remained the norm, limiting the opportunities for providers to discuss each prenatal topic in‐depth (ACOG, 2025). As a result, providers have expressed that there is not enough time to engage in meaningful shared decision‐making during the clinical visit (Légaré et al., 2008).

### 1.1 Medical decision aids

Medical decision aids (DAs) are tools that help people make informed, value‐driven healthcare choices, particularly in scenarios involving preference‐based decisions, when there is no “correct” medical option. In aneuploidy screening, where patients must weigh complex and often unfamiliar trade‐offs between risk, uncertainty, and unique personal values, DAs serve as a complement to clinical counseling. The most recent 2024 Cochrane update on DAs used in various healthcare decisions, from cardiovascular to mental healthcare, pooled 209 randomized controlled trials and over 107,000 participants, which reaffirmed and bolstered previous findings that DAs increase patient knowledge, improve risk perception, reduce decisional conflict, and support people making healthcare decisions that align with their unique values (O'Connor et al., 2009; Stacey et al., 2024). The use of DAs was associated with a 12‐point increase in knowledge (on a 0–100 scale), a nearly two‐fold increase in accuracy of risk perception (RR 1.94, 95% CI 1.64–2.34), and a greater likelihood of making healthcare decisions that prioritized participant values when values clarification components were specifically included. Importantly, the review found no evidence that DAs cause harm, including increasing anxiety or regret regarding decisions, and noted improvement in communication between providers and patients.

A 2021 scoping review, a type of systematic knowledge synthesis on a broad topic, by Yeşilçinar et al. specifically explored interventions aimed at improving informed decision‐making about prenatal screening and testing (Yeşilçinar et al., 2021). The review surmised that DAs delivered through diverse mediums, including written, video, digital, and in‐person, are effective in supporting deliberation and increasing knowledge, particularly when DAs include interactive or personalized elements.

Together, this growing body of literature reinforces the importance of DAs, specifically digital tools that incorporate risk visualization and values clarification, to enhance informed healthcare decision‐making in prenatal screening. Given time limitations in clinical practice and disparities in access to genetic counseling, DAs represent a promising strategy to ensure that all patients can make informed choices that reflect their preferences and values.

### 1.2 Preliminary research

**FIGURE 1:**
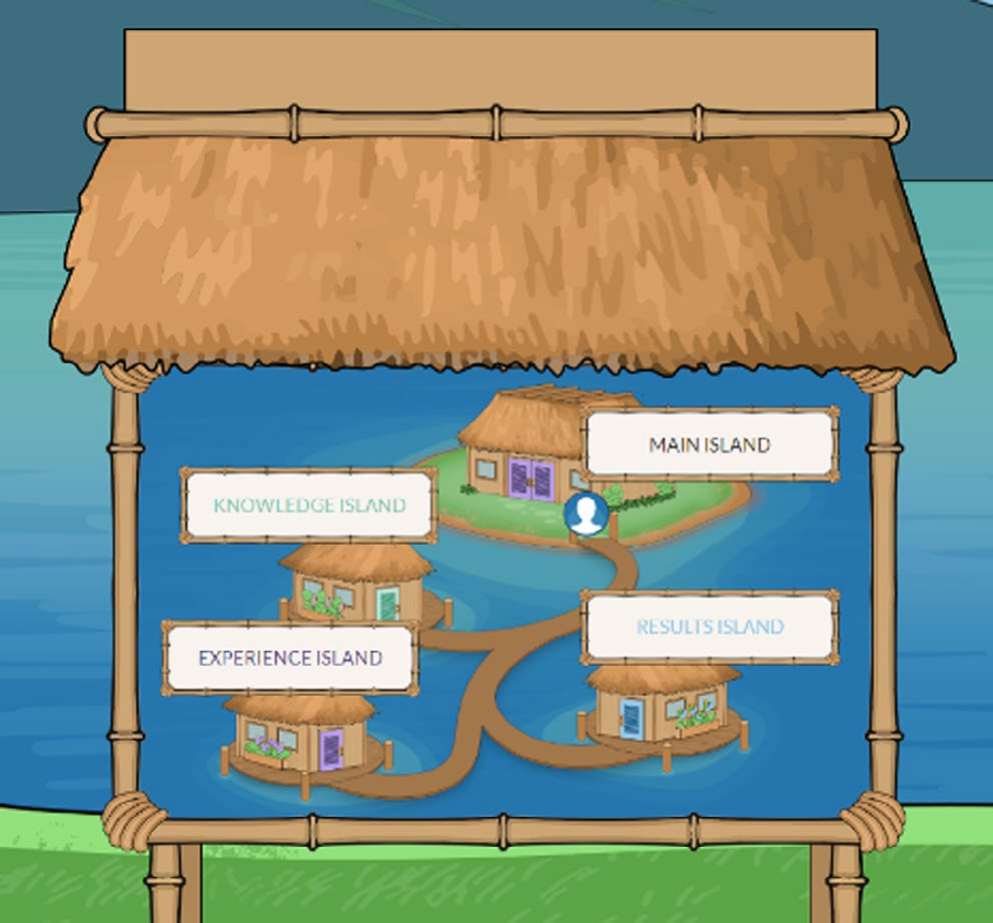
Screenshots of the map navigation page in the Meaning of Screening Game.

One promising approach to DAs is the use of interactive approaches to increase engagement with a decision aid tool. The Meaning of Screening, a web‐based app designed to support aneuploidy screening decision‐making, was created through interviews and iterative development with patients, providers, and experts. People use the app interface with a video game‐like navigation page to access information about aneuploidy screening (Figure 1). The Meaning of Screening app is being assessed in a national multisite randomized controlled trial (RCT) to determine its effectiveness on informational needs, preferences, and shared decision‐making of patients' aneuploidy screening decision compared to genetic counselors. Enrollment occurred at three sites in the United States: the University of Utah, the University of Alabama at Birmingham, and the University of North Carolina.

The app opens with the opportunity for participants to select their language (English or Spanish), select a personal avatar to follow them through the platform, and answer a set of values questions to consider their personal values around prenatal aneuploidy screening. They then move into the primary four “islands” (Figure 1), which a player could visit for information about aneuploidy screening (Knowledge island), read about other patients' experiences (Experience island), learn how maternal age impacts chance of a high‐risk Down syndrome result from their aneuploidy screening (Results island), and a central island where players could email themselves any useful information they wanted to keep from within the other islands, including their answers to the values clarification questions (Main island). The app's contents are written at an 8th grade reading level.

The development of this interactive app was informed by two studies. The first study developed and evaluated an earlier version of an interactive DA against a brochure‐based DA (Rothwell et al., 2019). In a randomized controlled trial, 73 pregnant women were randomized to either use a brochure or a game DA about aneuploidy screening. Participants who interacted with the app scored higher in knowledge than those who used the brochure (mean score 21.4 vs. 19.6, *p*‐values = 0.004, maximum score of 23), indicating that the app‐based decision tool was effective in educating pregnant individuals. The second study that contributed to the development of the current app was a qualitative study, which identified the aneuploidy screening decision‐making needs of minoritized pregnant individuals to ensure the updated game supports diverse populations (Riches et al., 2023). Study participants from three US health systems serving unique patient populations were interviewed about their decision‐making needs for aneuploidy screening. Several participants expressed that the information they were given prior to deciding was difficult to understand and receiving a high‐risk result was alarming because they didn't understand what this meant for them and their baby. The concept of “risk” was not adequately communicated. Study participants shared suggestions for improving the DA, including developing an interactive website or reading material, providing an explanation of test procedures, a list of conditions screened for, an explanation of probabilities, and stories from others who had been through the experience.

The goal of this current qualitative study is to summarize users' app experience, including how interaction with the online interactive DA impacted their decision‐making process and could be integrated into the clinical process.

## 2 METHODS

Ethical approval for this study was granted by the University of Utah Institutional Review Board (IRB_00101479) in July 2021. We conducted a qualitative analysis of the Meaning of Screening user experience using a constructivist paradigm. This paradigm is characterized by capturing an individual's experience by conducting research in their natural settings, using broad research questions and inductive analytic methods (Wainstein et al., 2023). Participants enrolled in the Meaning of Screening main trial were given the option to be interviewed about their experience with the game.

### 2.1 Participants

Only participants randomized to interact with the main Meaning of Screening app trial and who elected to be contacted for future interviews were eligible for an interview. The inclusion criteria for the main Meaning of Screening RCT included: the participant was at least 18 years old, spoke and understood English or Spanish, and was less than 15 weeks pregnant or a partner of an enrolled study participant. Individuals were excluded if they had undergone aneuploidy screening, had a high‐risk pregnancy due to family or obstetric history of chromosomal abnormalities, and/or had already met with a genetic counselor about aneuploidy screening. In addition to these criteria, the following additional inclusion criteria were included:

1. Made a decision to undergo or decline aneuploidy screening.
2. Spent at least 5 min on the app,
3. Interacted with the app ≤3 months prior to the interview date

The list was updated on a weekly basis as new participants met the inclusion criteria. Participants were all contacted by email or text, asking if they were willing to participate in an interview about their experience with the app. Participants who agreed to be interviewed underwent an additional consenting process. A member of the research team scheduled the phone interviews with consented participants. Participants were selected by sampling to achieve population representativeness. Out of the 45 participants contacted, 17 were interviewed. Recruitment for participation ensured saturation across educational attainment and study site. A $30 gift card was given to interviewees to compensate for their time.

#### 2.1.1 Participant interview guide

A semi‐structured interview guide was developed to explore study participant experiences with the app and how it may or may not have impacted their discussions with providers. Questions centered around the participant's technology experience, time constraints and distractions, opinions about the utility and relevance of the game, social influence, values and goals, and user experience with the game. Examples of questions include: Prior to visiting the meaning of screening web app, what did you know about prenatal genetic screening, such as cfDNA? What were your general impressions of the web app? How did the meaning of screening web app compare to health education websites or applications that you've used? (see Data S1).

#### 2.1.2 Data and data analysis

Interviews were video‐recorded and professionally transcribed, then reviewed for accuracy by a member of the research team. Inductive content analysis was conducted on the transcript data. A coding template was created from interview questions. Additional details were added to the codes based on the type of words used by interviewees to better capture details from their experience (Sandelowski, 2000). Interviews were coded simultaneously by three research team members, with discrepancies resolved through discussion. Codes with similar content were used to create categories and summarized. The results were then presented to the entire research team that incorporated two maternal–fetal medicine specialists, four researchers with qualitative and decision support expertise, and two research assistants. The research team recognizes that their positions as well‐educated academics may influence interpretation and interaction with study participants. However, our team provides a perspective that is both age and racially diverse, with gender identity that aligns with our cohort.

## 3 RESULTS

Seventeen participants were interviewed about their experiences with and opinions about the app. All participants were pregnant individuals; none of the partners volunteered to be interviewed. The participants' mean age at the time of enrollment was 30.4 years (SD = 5.5; see Table 1). Over half of the participants were non‐Hispanic White females (*n* = 10), had a high level of education (13 with an Associate's degree or higher), and had heard about aneuploidy screening prior to interacting with the app (*n* = 13).

**TABLE 1:** Descriptive information about the study participants interviewed (*n* = 17).

Site	Site 1	5 (29.41%)
	Site 2	5 (29.41%)
	Site 3	7 (41.18%)
Age	Mean = 30.41	SD = 5.52
Sex	Female	17 (100%)
Race	White	10 (58.82%)
	Black	3 (17.65%)
	Asian	3 (17.65%)
	Other	1 (5.88%)
Ethnicity	Hispanic	2 (11.76%)
	Not Hispanic	15 (88.24%)
Education	< High school	1 (5.88%)
	High school/GED	2 (11.76%)
	Some college	1 (5.88%)
	Associates/vocational	1 (5.88%)
	College graduate	4 (23.53%)
	Professional/graduate degree	8 (47.06%)
Household Income	<$24,999	2 (11.76%)
	25,000–50,000	3 (17.65%)
	50,001–100,000	2 (11.76%)
	100,001–150,000	5 (29.41%)
	150,000+	3 (17.65%)
	Not disclosed	2 (11.76%)
Previously heard about aneuploidy screen	Yes	13 (76.47%)
	No	4 (23.53%)

Three major categories related to participants' experiences were identified through inductive content analysis: *Impact, User Experience*, and *Integration into Genetic Counseling and the Clinical Environment*.

### 3.1 Impact

There were multiple impacts of the app: Knowledge and understanding, values clarification, decision‐making factors, and shared decision‐making. Participants expressed the impact of the app on their knowledge and understanding, contributing to their final prenatal screening decision, in multiple ways. Several participants stated that the app clarified their options, which in turn helped them make a decision. About half noted that the app supported their preexisting decision, and the remaining participants expressed that the app did not impact their decision. One participant used the app to convince her husband that they needed to do aneuploidy screening.

#### 3.1.1 Greater knowledge and understanding

Most participants stated that they had heard about aneuploidy screening prior to using the app, but when asked what they knew, few had accurate knowledge. Some individuals specifically noted that they were unaware that it was not diagnostic. Three participants indicated their healthcare provider had discussed screening with them, and another learned about it in medical school. However, there were several participants who referred to it as a “gender test” (*n* = 3) and three others knew of the test but were unfamiliar with the term cfDNA test.

I just knew that when you get pregnant you need to do some genetic testing. I had no idea what that entailed or how evasive [invasive] it was or even whether there was any risk associated with it or the fact that some women choose not to do it. And so in my mind it was just like that's part of getting pregnant. You do the tests that they ask you to do (Participant 8)

**FIGURE 2:**
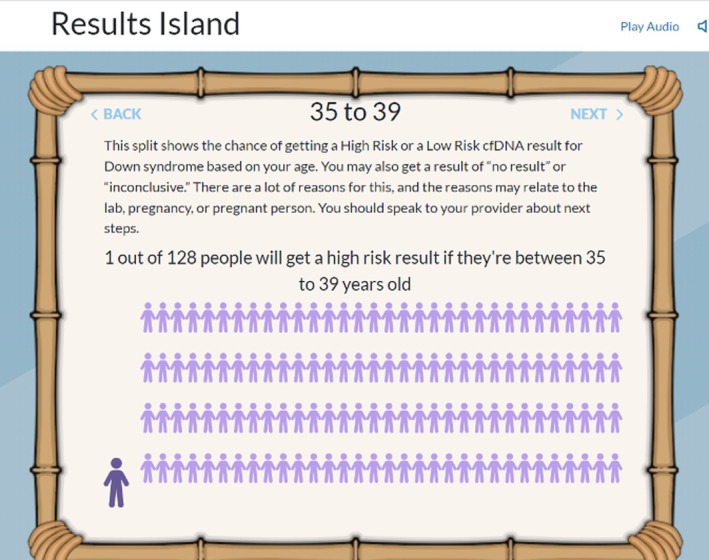
An image from Results island demonstrating risk.

A majority of participants valued learning about the different types of tests and the difference between screening and diagnostic testing, while others found the presentation of the statistics and interpretation of results to be more helpful for them (Figure 2). Only one person found that it was most helpful to hear about other people's experiences.

I think it just gave information. I don't think it really convinced us one way or the other. I think the experience I learned was helpful in that manner. It wasn't very biased. It just gave the differing views of why you might want to or not want to know. And so I don't think it pushed us one way or the other. (Participant 7)

It's actually not a diagnostic test, it's just a screening to show if you're higher risk or lower risk for certain things. So, I think that that's really helpful for people to know is that it's not a certainty, it's just a screening for, I guess you'd call it certain markers, but the difference between a screening and a test. (Participant 2)

#### 3.1.2 Values clarification

**FIGURE 3:**
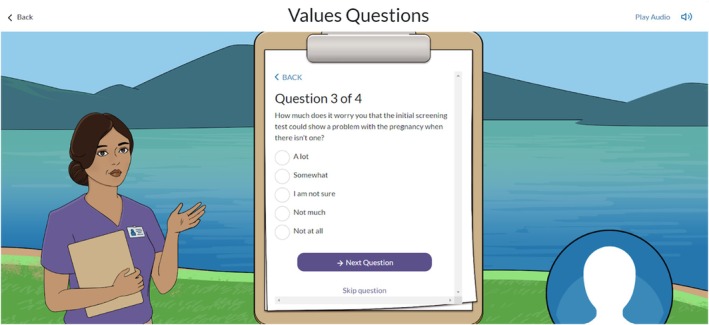
Screenshots of one of the values questions in the Meaning of Screening Game.

Although no one specifically called out the values questions (Figure 3) as providing helpful information, when directly asked about them, users indicated that the values questions helped them make a decision by preparing them for a potential high‐risk result, introducing them to new perspectives, and allowing them to frame subsequent information in a different way.

I think that [the values questions] was a good way to start because it made me think about everything that I was reading a little deeper and it helped me come to my conclusion not to do anything because at the end of the day, to me it wouldn't important to me, but at the same time it wouldn't change my decision in any type of way, which is why I came to the conclusion that I didn't want to do any testing. (Participant 14)

#### 3.1.3 Decision‐making factors

When considering the most important factor in their aneuploidy screening decision, many participants called out their personal values, planning for the future in the case of a positive screen, life experiences with special needs children or children with behavioral problems, spirituality, and cost.

I guess my personal values and just wanting to be able to prepare for the future the best way possible. I think knowledge is power and if you know that there's a high chance of your baby having a health problem, I think it's important to know so that you can be adequately prepared. (Participant 9)

Additionally, half of the participants indicated that they did not seek out further information, while the other half said they searched the internet and/or had a discussion with their healthcare provider but neither swayed their decisions.

I think it just kind of helped solidify our decision because we had kind of decided before the study to do the testing, but after reading through the information and accessing the web app and talking with my husband, we both had decided that we wanted to go ahead with it and that it kind of solidified our decision to continue with the testing. (Participant 6)

#### 3.1.4 Shared decision‐making

Participants also expressed the impact of the game on conversations they had with their partner or healthcare provider. A few people indicated that they discussed the information from the app with their prenatal care provider in order to further explore personal risk and testing options. Some of those who chose not to discuss the information with their prenatal care provider indicated that they made that choice because they had no additional questions after using the game or because they had not been back to see the provider since using the app.

I talked about all the information that I actually learned from the app [game] because I didn't make the decision on my own as well. I also educated my husband on what I knew for us to both make a decision together. (Participant 1)

I definitely got more information, and I had a good communication with my doctor regarding what would be best for me. And that played a role in deciding what I wanted to do regarding screening. (Participant 11)

Expressing thoughts about how the app might impact others' shared decision‐making, participants noted that it would improve a patient's prenatal provider visit by providing time to process the information and be more prepared in advance, helping them to work through their thoughts in advance, and giving the patient more agency in pregnancy‐related decisions.

I think it would foster a more meaningful conversation, especially because providers are busy these days…. [but] if you prescribe it and they don't have a computer at home or a way to access it, it might be a little disheartening or kind of put off‐ish (Participant 6)

### 3.2 User experience

#### 3.2.1 Game navigation

**FIGURE 4:**
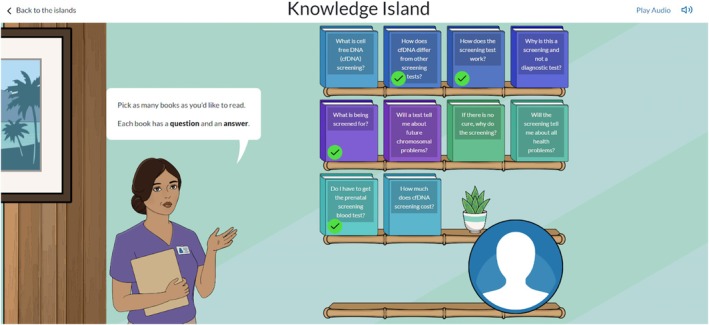
Screenshots of Knowledge Island in the Meaning of Screening app.

When asked about their initial impressions and how they felt about navigating the app, most of the participants felt that the app was easy to use, providing “bite‐sized” pieces of information (Figure 4) and a comfortable flow across topics. A few people noted that the app was visually pleasing, which helped with navigation and feeling comfortable using it.

…I just thought it [the game] was so easy to use and it was such a… is a good tool. It provides you with the information that you're wanting to know without just seeing it on a piece of paper that can be very confusing. It's very simple to understand, so I would definitely recommend it to somebody else to look at. (Participant 9)

Overall, most users did not express any difficulty using the app. However, three participants mentioned that it took them several clicks to understand how to navigate the game‐like interface and another mentioned they felt the app was a little slow or “clunky,” particularly in the opening screens.

…it was kind of hard to get started. I didn't really know what I was supposed to do. But yeah, I mean once you trial and error it enough, it was easy. (Participant 15)

Several participants highlighted that they liked the ability to choose what information to look at for a user‐specific experience. On the other hand, several participants had no previous experience with interactive games and chose to compare the app to their usual source of information, mainly reading papers or websites. They expressed that the app was more engaging and the information was easy to locate. However, a couple of participants stated they prefer to read educational material over receiving it through the interactive app.

I think that the only thing is that you had to walk through the steps in order to get the information. And I think for somebody who likes to gather that information quickly, it could be nice to have another format available for someone who just wants to read through the information. (Participant 7)

I liked being able to refer back to the email probably more than I did the app [the game], honestly, because it was more like a book. (Participant 14)

However, some noted that the app could be difficult for people with a lower literacy level or no technology access; it could create an opportunity for people to stress about the potential results even earlier, or the app could be viewed as having an agenda and encouraging a particular choice.

#### 3.2.2 Influence of professional background

Several participants expressed that their background played a role in the opinion they initially formed about the app, aneuploidy screening, and their ultimate choice. For instance, because of her professional role, one participant knew that she wanted early screening because she wanted to know if she would have a child with a chromosomal abnormality as early as possible.

Yeah, so I am a clinical mental health counselor, and I work with kids with behavioral problems. And I see the struggle that folks deal with day in and day out… I am pretty firm in my belief that I didn't want to bring a kid into the world to struggle through that. And I didn't think that I had the capacity to care for a child who couldn't care for themselves independently throughout the entire course of their life. (Participant 14)

Three participants had a medical background, which also influenced their experience with the app.

To be honest, when I first opened it up, I thought it looked somewhat elementary. And I do think that's because I have some medical background. But once I started using it, I felt like it was very easy to navigate. It wasn't as long as I thought it was going to be. I felt like it was concise and simple and easy to look at, especially when I started thinking about the patient population that doesn't work in a hospital and how it probably was really simple and easy to understand. …Actually, I liked it better than I thought I would, to be 100% honest. (Participant 9)

Many of the participants specifically indicated that the app would be helpful to people regardless of their background or prior knowledge.

#### 3.2.3 Referencing app information

**FIGURE 5:**
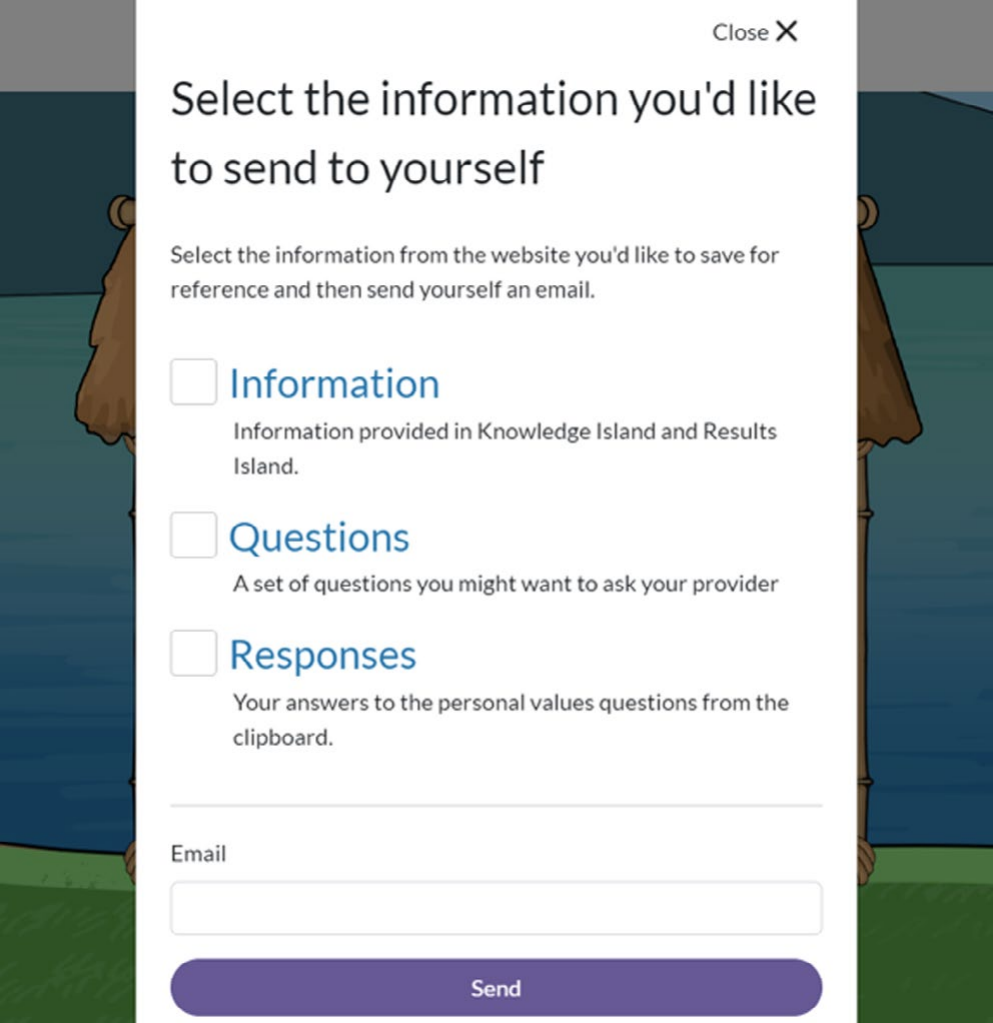
Screenshot of the Main Island Meaning of Screening game.

One feature of the app is the ability for the user to email themselves the results of their values questions, the informational details from the Knowledge Island and Results Island, and/or a list of useful questions for their provider about aneuploidy screening (Figure 5). Four of the seven participants who opted to email themselves this information noted that they also referred back to that email for information: “I like having a resource that I can refer back to.” One user indicated that she did not refer to the email, but has revisited the app on multiple occasions, whereas another took screen shots that she said she referred back to: “And I also took pictures of it on my phone.” Alternatively, one participant mentioned that they didn't email themself because they knew they could refer to the website: “I did not email it to myself. Because I knew I had this website.”

Additionally, most of the participants discussed some aspect of the app with their partner, mother, or sister. The discussions described ranged from a summary of the app to the difference between screening and diagnostic examinations. Only a few people mentioned that they discussed the app information with their prenatal care provider, which is discussed further in the Impacts categories.

I had talked to my family about it. They were all asking like, oh, are you going to find out the gender? And I was like, yes, we're going to find out the gender, but also we're doing this NIPT testing which can screen for potential genetic abnormalities or chromosome abnormalities. And so, I kind of talked to them about it and how we kind of learned more about it through this process. (Participant 6)

### 3.3 Integration into genetic counseling and the clinical environment

Participant responses regarding how the app could be used in a clinical environment, as a replacement for pre‐test genetic counseling or alongside genetic counseling, were varied. Some participants recognized that patients may have personal reasons for choosing to meet with a genetic counselor, including a family history or advanced age, as an incentive to get more information than the game provided. Some participants indicated that their preference for meeting with a genetic counselor was to ask questions in real‐time. In contrast, some participants shared that they felt the app would work well as a standalone educational tool, to use at their own convenience because the cost is lower, and, for some, it would satisfy their need to not interact with people.

Some people is not good with people, some people is. (Participant 12)

The remaining participants gave more nuanced answers, stating they both liked the time flexibility of the app and desired a conversation with a person. Others expressed that the app was a great source of pre‐visit information, which helped to prepare for meeting the genetic counselor.

I did this before I actually met with the genetics counselor, so it kind of gave me an idea of what to expect. (Participant 5)

Participants generally indicated that they would be willing to spend “as much time as needed” or “about an hour” educating themselves on aneuploidy screening outside of the clinic, indicating a strong drive to understand the tests they are agreeing to complete. However, the participants interviewed spent an average of 15 min interacting with the Meaning of Screening app. One participant shared that they felt the app was time‐consuming.

## 4 DISCUSSION

Deciding whether to accept or decline aneuploidy screening and further diagnostic testing is a complex process influenced by personal and partner knowledge, values, and beliefs. This decision depends on social and familial acceptance, the willingness to care for a potentially affected child, and perspectives on pregnancy termination, both personal and societal (Ravitsky, 2017; Ravitsky et al., 2021). The goal of this study was to assess how an online gamified decision aid, accessed as part of a larger clinical trial, impacted users' decisions regarding aneuploidy screening.

DAs have shown time and time again to be powerful tools that improve decision quality for patients facing difficult healthcare decisions where there is no clear best option. Our study participants indicated that the Meaning of Screening app can provide the education needed to support the aneuploidy screening medical decision‐making process and allow for personal autonomy to make that decision, a key goal in healthcare decision‐making. Autonomy in reproductive decision‐making requires patients to be well informed of their prenatal testing choices enough to ensure their choice aligns with their personal values and attitudes toward the process and potential outcomes. Healthcare professionals play a crucial role in providing accurate, thorough, and unbiased information about available options. However, during each prenatal visit, the provider has a number of competing clinical education needs (Dyer et al., 2018; Gammon et al., 2016). When limited time doesn't allow for proficient education in the clinic, it is important that patients can obtain accurate information through other means and be encouraged to integrate this knowledge with their values when considering the potential implications of undergoing prenatal screening. Some of our study participants highlighted the app's convenience, which may promote autonomy by allowing them to learn at their own pace and when their schedule permits.

Professional guidelines agree that patients should receive counseling prior to undergoing aneuploidy screening (ACOG, 2020; Dungan et al., 2023; Hui et al., 2023), with the most effective approach to education about this screening being one‐on‐one genetic counseling (Fonda Allen et al., 2016). Genetic counseling provides families with both needed information and psychological discussions that could reduce anxiety and potentially ill‐informed decisions for termination of pregnancy (Lee & Chan, 2023; National Society of Genetic Counselors' Definition Task Force et al., 2006). However, in the United States, due to the limited number of genetic counselors (Villegas & Haga, 2019), the timing at which the aneuploidy screening is completed (typically 10+ weeks' gestation), and the recommendation that all patients, regardless of risk, be offered aneuploidy screening, this level of counseling is not feasible. This clinical specialty may not be available to everyone. As a result, prenatal care providers are frequently the ones offering aneuploidy screening to patients with genetic counseling only offered after a high‐risk result or to patients with a family history of chromosomal abnormalities (Eltabbakh et al., 2024). Consequently, many patients are making decisions about screening with incomplete or inaccurate information (Johnson et al., 2024). The Meaning of Screening app was found to be a scalable and patient‐accepted method that may also enhance shared decision‐making with a prenatal provider or genetic counselor.

Providing a tool, such as The Meaning of Screening, prior to the prenatal visit when screening is ordered or the scheduling of a post‐test genetic counseling visit could provide important information for patients at multiple useful timepoints. For instance, if the tool is provided immediately after they receive their high‐risk result but before they are able to speak with the genetic counselor, it could decrease stress and anxiety prior to the counseling visit and allow the counseling visit to be more productive because much of the educational content has already been covered. In this study, some study participants expressed that the app provided valuable pre‐visit information, which helped them prepare for discussions with their genetic counselor. The authors are currently completing interviews with genetic counselors to explore these possibilities.

Another important element to consider is that, in contrast to written information or other computer applications, gamified education is not a one‐time dissemination of information (Brown et al., 1997). Games allow users to personalize their learning experience by offering different simulated roles and choices to explore health care decisions in an engaging and interactive manner, providing a large amount of didactic content through unlimited opportunities to return to the app and explore different results or answers to different questions (Sicart, 2011; Zagal, 2011). These kinds of features have been found to significantly increase users' attention and retention of knowledge compared to brochures or pamphlets (Arif et al., 2024; Kozma, 1991; Nylén‐Eriksen et al., 2025). Through game technology, these opportunities to repeat and explore ranges of options can improve decision quality. Several participants in this study said they continued to interact with the app after completing their research participation in the main trial, referencing either the app or the informational email sent from the app. Many also shared information they learned from the app with their partners, friends, and other family members. This finding is particularly notable since a primary goal of providing aneuploidy screening education is to improve the shared decision‐making process, either through interactions with their provider or partner. A web‐based gamified app like the Meaning of Screening could be an efficient method of transmitting the knowledge participants seek during routine clinical appointments or in conjunction with genetic counseling for “high‐risk” results.

## 5 STRENGTHS AND LIMITATIONS

The strengths of this study include the representativeness of the interviewed population, who were enrolled at three healthcare systems across the United States. However, the study is limited by the overall higher level of education of the participants interviewed and the use of the app within a larger clinical trial environment. We do not yet know how patients who have access to the app will interact with it outside of a research setting. Those who participate in research may not represent the general public. In particular, the subgroup that agreed to complete interviews had a high level of education (70% were college educated or higher).

## 6 CONCLUSION

Results from this study indicate that a web‐based interactive app that supports informed decision‐making about aneuploidy screening, such as the one assessed here, could increase patient autonomy by (1) improving patient knowledge about aneuploidy screening and the impact of their decision prior to their prenatal clinical visit, (2) improving decisional quality for the patient through integration of values‐based questions, and (3) engaging participants with the material better than a standard educational brochure or website. The Meaning of Screening app is a tool that could be easily integrated into clinical environments to aid in patient education.

## AUTHOR CONTRIBUTIONS

**Naomi O. Riches:** Data curation; formal analysis; supervision; writing – original draft; writing – review and editing. **Erin P. Johnson:** Conceptualization; data curation; formal analysis; project administration; supervision; writing – original draft; writing – review and editing. **Akila Subramaniam:** Project administration; supervision; writing – review and editing. **Neeta L. Vora:** Project administration; supervision; writing – review and editing. **Kimberly A. Kaphingst:** Conceptualization; methodology; writing – review and editing. **Jenna Junge:** Data curation; writing – original draft; writing – review and editing. **Malique Valle:** Data curation; writing – review and editing. **Erin Rothwell:** Conceptualization; methodology; writing – review and editing.

## CONFLICT OF INTEREST STATEMENT

Naomi O. Riches does not have any conflicts of interest to disclose. Erin P. Johnson does not have any conflicts of interest to disclose. Akila Subramaniam does not have any conflicts of interest to disclose. Neeta L. Vora does not have any conflicts of interest to disclose. Kimberly A. Kaphingst does not have any conflicts of interest to disclose. Jenna Junge does not have any conflicts of interest to disclose. Malique Valle does not have any conflicts of interest to disclose. Erin Rothwell does not have any conflicts of interest to disclose.

## ETHICS STATEMENT

Ethical approval for this study was granted under the University of Utah Institutional Review Board (IRB_00101479).

## AI STATEMENT

AI was not used in the writing of this manuscript.

## PATIENT CONSENT STATEMENT

Study participants completed an informed consent prior to study participation.

## Supporting information


Data S1.

## Data Availability

Data used in this manuscript will not be made available because the study participants did not give written consent for their data to be shared publicly.
