# Supplementary material for: The Meaning of Screening: Exploring user experience of an aneuploidy screening educational game
Source: J Genet Couns. 2026 Feb 18;35(1):e70183. doi: 10.1002/jgc4.70183 (PMC12915519; doi:10.1002/jgc4.70183)
Supplement: Supplementary file 1 — Data S1. [file JGC4-35-0-s001.docx]

**Meaning of Screening Patient Interviews**

**Purpose**: to identify how the game impacted information needs, preferences and shared decision-making. To provide a more nuanced assessment of how the patient-provider interactions may differ because of game intervention. How did the game impact the decision-making process?

**Script**

Thank you again for agreeing to share your thoughts. As you’ve heard, during this interview, we will ask you questions about your experience with prenatal genetic screening and the Meaning of Screening website, also called the “web app” in this interview. There are no right or wrong answers to these topics and different people can have different thoughts and beliefs. We are interested in what you think.

This interview will last about 20 minutes, and my role will be to ask questions and listen to your answers. Please remember that you may choose not to answer any question and you can stop participating at any time.

I would also like to remind you that this interview will be recorded, and that the recording will be heard only by the research staff on this project, or by a professional transcriptionist. We will remove any information that could identify you after the interview. We will keep all the information you tell us during this interview confidential and will only use your answers for research purposes. Any questions before we get started? [**Answer questions**]

I will start the recording now. [**Start recording**]

Opening

Early in your pregnancy you were asked to visit a web application called Meaning of Screening [*show image of opening screen*]. This decision-making tool opens with questions about your values and then provides education about prenatal genetic screening, also known as cfDNA, through different islands of a resort: Knowledge Island, Results Island, Experience Island and Main Island. As we continue the interview, please think about your experience with this web application.

1. Prior to visiting the Meaning of Screening web app, had you heard about prenatal genetic screening (aka cfDNA)?
   1. If so, what did you know about it?
2. Do you remember visiting the Meaning of Screening web app? What were your general impressions with the web app?

Technology Experience

1. Thinking about other health education websites or applications that you’ve used, how did your experience with the Meaning of Screening compare?
2. When you were using the Meaning of Screening web app, how easy was it to use and navigate? What about the web app kept you wanting to use it?
3. What was the most challenging aspect of using the web app? What about it was not very clear or engaging?
4. The Meaning of Screening web app included five different parts [*show image of map with components]*: 1) the values questions at the beginning, 2) Knowledge Island, 3) Experience Island, 4) Results Island, and 5) the Main Island. Do you remember which parts you visited? What do you remember about them?
   1. Did you email the information to yourself from Main Island? If so, did you refer back to that email?

Time constraint and distraction

1. Meeting with a genetic counselor takes about 1 hour. Another option is to use the Meaning of Screening app for as little or as long as someone wants to.
   1. What option would you prefer and why?
   2. What do you think other people might prefer?
2. How much time are you willing to spend educating yourself, outside of your clinic visit on something like the Meaning of Screening website, to help you make a health decision?

Relevance/Utility:

1. Did the Meaning of Screening web app help you understand your prenatal genetic screening options?
   1. if so, how? if no, tell me why not.
2. What information provided by the Meaning of Screening web app was most helpful to you?
3. Did you learn new information from the app?
   1. if yes, what was it?
4. Is cfDNA (prenatal genetic screening) different than what you thought it was? How? (skip if they’ve already answered this in earlier questions)
5. What role did the web app play in your decision about prenatal genetic screening?
   1. Did it help you make a decision about ordering cfDNA? Why? How?
6. Did you discuss any of the information from the app with your healthcare provider?
   1. if yes, how? if no, why not?

Social Influence

1. Did you talk with anyone else about the Meaning of Screening app?
   1. What did you talk about?
2. Did you talk about the app with your partner? (skip if answered previously)
   1. What did you talk about it?
3. Did you look for additional information anywhere else? How did that information impact how you made your decision?

Values and Goals

1. [*show image of the opening values questions*] At the beginning of the Meaning of Screening web application you were asked to answer a few questions about your values. How did those questions help you think about your final decision on prenatal genetic screening, both with the app and with your subsequent conversation with your provider?
2. What was most important to your final decision (e.g., personal values, experience with family, spirituality, finances, external support)?

Introducing/Using the tool:

1. The MoS website is an example of an online web application to help patients make a health decision. This type of tool is often used to provide information about health topics that there may not be time to fully discuss during a visit with your health care provider
   1. How would you feel about your medical provider asking you to use the Meaning of Screening web app before an appointment?
   2. What do you think would be the benefits to all newly pregnant patients being prescribed to use this web app?
   3. What are some reasons you might think it wouldn’t be beneficial?
   4. When would you prefer to use the Meaning of Screening app?

i. emailed to you before your visit

ii. Before your appointment in the waiting area?

iii. In the appointment with your health care provider?

1. Would you recommend the Meaning of Screening app to another patient? Why or Why not?

Do you have any other comments about using the web app for pregnant people that we did not already discuss?
